# Amperometry approach curve profiling to understand the regulatory mechanisms governing the concentration of intestinal extracellular serotonin

**DOI:** 10.1038/s41598-024-61296-9

**Published:** 2024-05-07

**Authors:** Mark S. Yeoman, Sara Fidalgo, Gianluca Marcelli, Bhavik Anil Patel

**Affiliations:** 1 School of Applied Sciences, University of Brighton, Huxley Building, Brighton, BN2 4GJ UK; 2 Centre for Lifelong Health, University of Brighton, Huxley Building, Brighton, BN2 4GJ UK; 3 School of Engineering, University of Kent, Jennison Building, Canterbury, CT2 7NZ UK

**Keywords:** Serotonin, Colon, Ileum, Enterochromaffin cell, Autoreceptors, Transmitter, Regulation, Bioanalytical chemistry, Peripheral nervous system

## Abstract

Enterochromaffin (EC) cells located within the intestinal mucosal epithelium release serotonin (5-HT) to regulate motility tones, barrier function and the immune system. Electroanalytical methodologies have been able to monitor steady state basal extracellular 5-HT levels but are unable to provide insight into how these levels are influenced by key regulatory processes such as release and uptake. We established a new measurement approach, amperometry approach curve profiling, which monitors the extracellular 5-HT level at different electrode–tissue (E–T) distances. Analysis of the current profile can provide information on contributions of regulatory components on the observed extracellular 5-HT level. Measurements were conducted from ex vivo murine ileum and colon using a boron-doped diamond (BDD) microelectrode. Amperometry approach curve profiling coupled with classical pharmacology demonstrated that extracellular 5-HT levels were significantly lower in the colon when compared to the ileum. This difference was due to a greater degree of activity of the 5-HT transporter (SERT) and a reduced amount of 5-HT released from colonic EC cells. The presence of an inhibitory 5-HT_4_ autoreceptor was observed in the colon, where a 40% increase in extracellular 5-HT was the half maximal inhibitory concentration for activation of the autoreceptor. This novel electroanalytical approach allows estimates of release and re-uptake and their contribution to 5-HT extracellular concentration from intestinal tissue be obtained from a single series of measurements.

## Introduction

Within the intestinal mucosal epithelium resides enterochromaffin (EC) cells, which release serotonin (5-hydroxytryptamine; 5-HT) following either chemical or mechanical stimulation^1–3^. EC cells serve as epithelial transducers between the lumen and the underlying neuronal and glial network of the enteric nervous system^4,5^. EC cells play an important role in regulating propulsive motility patterns^6–9^, barrier function^10^ and immune system^11,12^ through the release of 5-HT. Extracellular 5-HT concentrations are tightly regulated through reuptake via the serotonin transporter (SERT) located on the EC cell and surrounding epithelial cells known as enterocytes ^4,13^. Pharmacological studies of the EC cells in various animal models and humans have also identified the presence of 5-HT_4_ and 5-HT_3_ autoreceptors on EC cells, which also can regulate extracellular 5-HT levels, through their effects on 5-HT release^14–16^.

Electroanalytical techniques have been widely used to monitor levels of 5-HT from ex vivo intestinal tissue using either amperometry^17–19^ or fast scan cyclic voltammetry^20^. Such studies have provided the ability to understand the relationship between 5-HT and motility patterns^8,9,21,22^ as well as providing key insights into how extracellular 5-HT levels are altered in the presence of different nutrients^23,24^, disease^25–27^ and ageing process^28^. However, with all ex vivo intestinal tissue measurements conducted to date, the steady state basal levels of 5-HT that are monitored are due to the media flow through the organ bath providing a constant mechanical stimulus to the EC cells. This makes it challenging to understand how different regulatory processes (release and re-uptake) contribute the observed steady-state basal levels. This is less of a challenge for the electrochemical measurement of neurotransmitters from single cells, brain slices and in vivo measurements in the brain. Here dynamic rather than steady state changes in the current are observed following spontaneous or evoked neuronal activity^29–32^, which can be related to release and re-uptake processes which influence the extracellular neurotransmitter concentration.

At present no methodological approach provides the means to be able to decouple the contribution that key regulatory processes, such as release and re-uptake, play in defining the observed steady-state basal extracellular concentration of 5-HT during measurement from ex vivo intestinal tissue. Within this study we developed a simple approach known as amperometry approach curve profiling to monitor the extracellular levels of 5-HT at different electrode-tissue (E–T) distances. By conducting measurements at different E–T distances, the concentration profile of the basal released 5-HT from the intestinal epithelium could be sampled, which provides scope through analysis to understand the contribution that release; reuptake and autoregulation can have on extracellular levels of 5-HT. We have utilised ex vivo intestinal segments from murine ileum and colon in combination with a range of pharmacological agents to validate the suitability of this technique in providing information on the regulation of 5-HT extracellular levels and to provide dose–response relationships for 5-HT on the SERT and inhibitory autoreceptors.

## Results

### Sensitive and stable detection of 5-HT from isolated intestinal tissue

**Figure 1:**
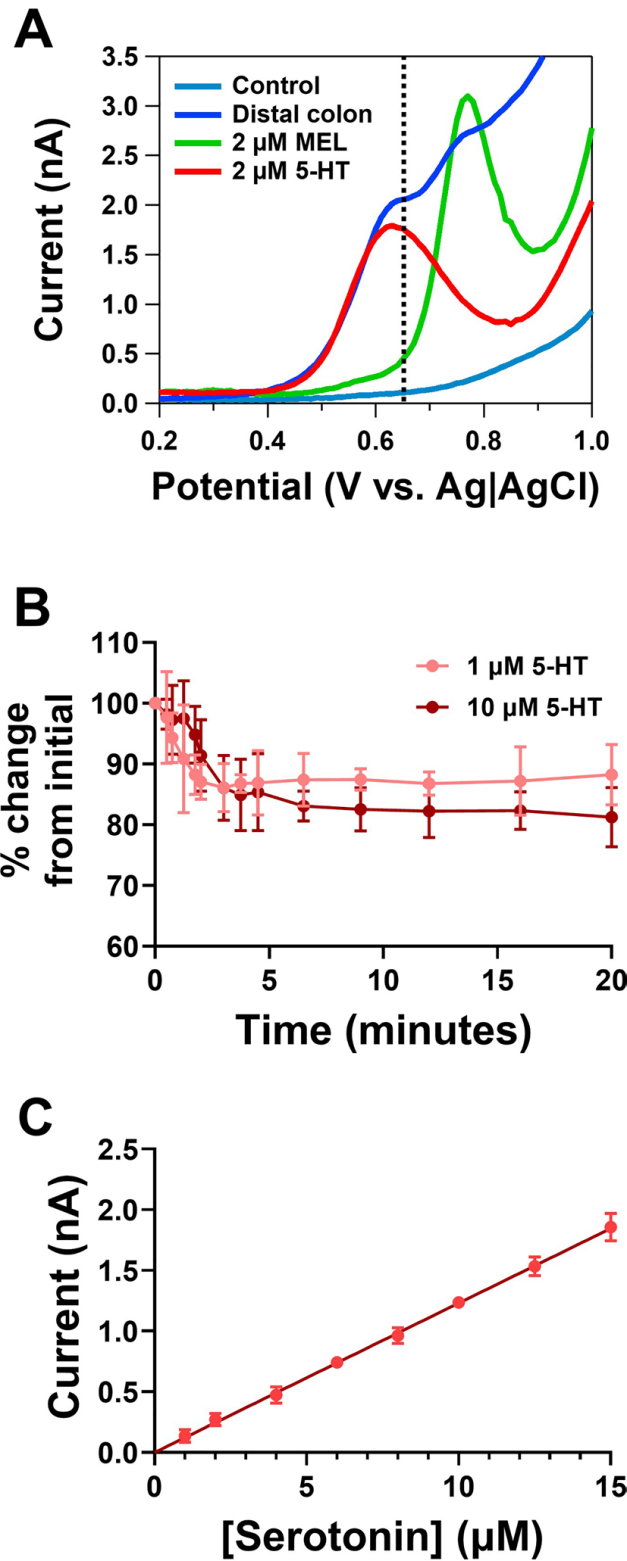
Electroanalytical performance of the boron-doped diamond (BDD) microelectrode for detection of 5-HT release from intestinal tissue. (**A**) Differential pulse voltammogram from distal colon mucosal tissue, showing the presence of two oxidation peaks, which corresponded to 5-HT and melatonin. Amperometric recordings at + 650 mV vs Ag|AgCl, provide selective detection of 5-HT overflow (see dotted line). (**B**) Stability of the BDD microelectrode for measurement of 1 and 10 µM 5-HT in Krebs buffer (n = 6). (**C**) Calibration plot of 5-HT on the BDD electrode (r = 0.99, n = 8).

Figure 1 shows the electrochemical performance of the BDD electrode for monitoring 5-HT release. Figure 1A shows a differential pulse voltammogram taken over the distal colon tissue at an E–T distance of 100 µm, demonstrating two clear peaks under the potential window investigated. The first peak around + 650 mV corresponded to 5-HT. The dashed line at + 650 mV in Fig. 1A represents the voltage utilised for amperometric measurements, which solely allows for the detection of 5-HT. The second peak at + 800 mV was identified as melatonin. Melatonin has been previously shown to be released from EC cells from various species, however no other easily oxidisable compounds were observed^3,33^. These results also validate those observed using a chromatography method where extracellular fractions from isolated distal colon mucosa tissue were shown to only contain 5-HT and melatonin^34,35^.

Figure 1B shows the stability of the BDD electrode for monitoring physiological concentrations of 5-HT (1 and 10 µM 5-HT) for 20 min, the typical time period of our recordings. Various studies have shown that oxidative by-products of 5-HT are known to have a strong affinity for the electrode causing fouling^36–38^. Although there is an initial drop in the sensitivity of the sensor between 0 and 2 min, there is no significant diminishment of the current between 3 and 20 min in the concentrations investigated, where approximately 90% of the signal is intact. During our recordings, BDD electrodes are pre-fouled in 10 µM 5-HT for 5 min so that stable recordings can be obtained for the duration of biological measurements (not greater than 15 min). Finally, the BDD electrode has good sensitivity and excellent linear range for the measurement of 5-HT from EC cells (Fig. 1C), and thus is fit-of-purpose.

### Amperometry approach curve profiling and the mathematical model utilised to define the parameters regulating extracellular 5-HT concentration

**Figure 2:**
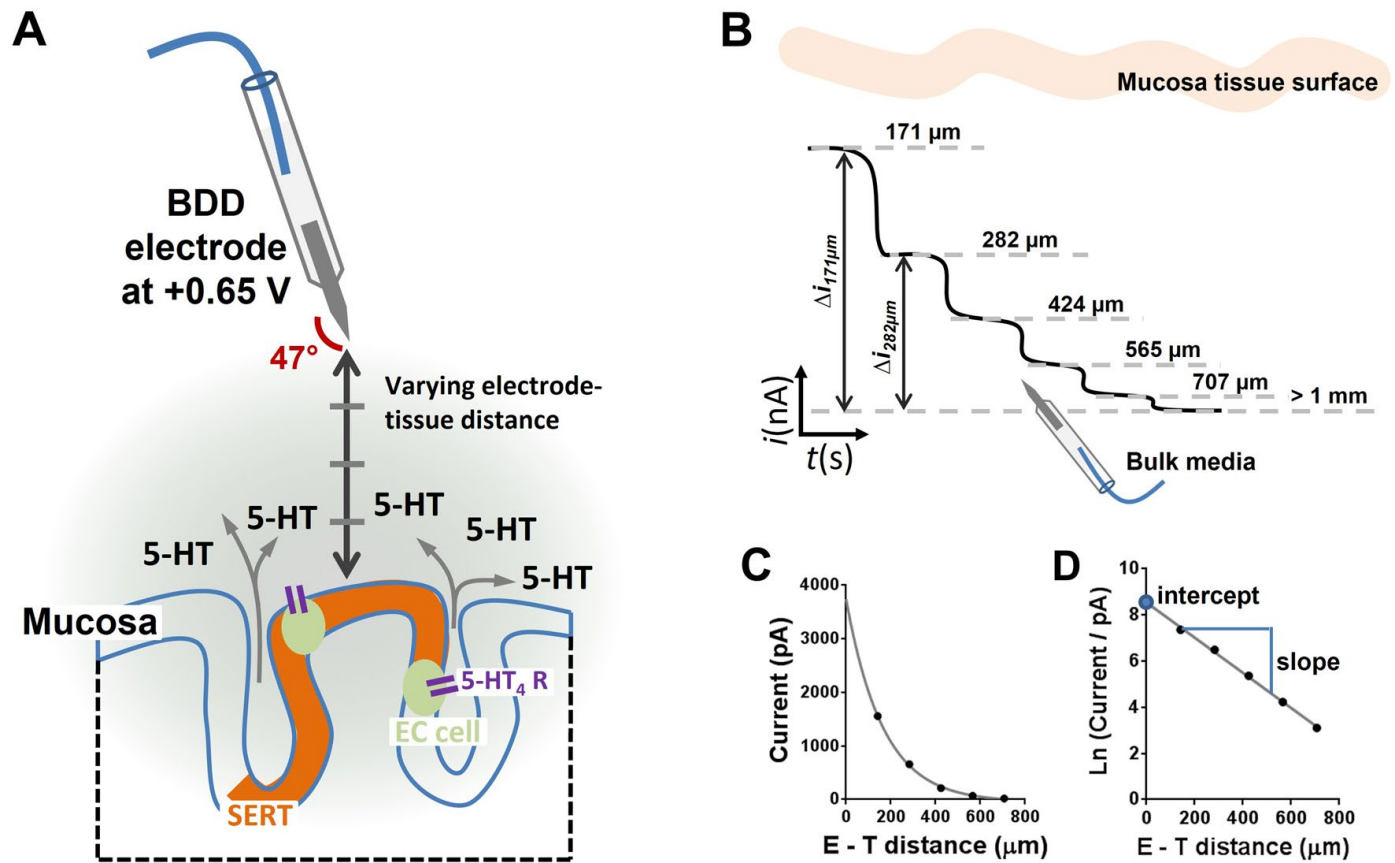
Amperometry approach curve profiling methodology. (**A**) Measurement protocol from mucosal tissue, indicating the process by which amperometry approach curve profiling recordings are conducted. (**B**) Example of an expected current trace where recording at each E–T distance is carried out every 40 s. (**C**) The resultant current difference at each step is plotted at the various E–T distances. (**D**) The natural log of the current is obtained and plotted against the E–T distances, where the intercept is used as a marker of release and the slope as a marker of reuptake.

Distance values from the tissue to the electrode measured using the micrometer scale on the manipulator were corrected for the manipulator angle of 47°, thus for measurements carried out at manipulator scale readings of 1000, 800, 600, 400 and 200 µm, the actual E–T distances were 707, 565, 424, 282, 141 µm (Fig. 2A). These specific distances were chosen as the error in the current increased due to the influence of the flow rate (convection) at E–T distances greater than 750 μm and tissue surface geometry effects at E–T distances less than 100 μm^39^. For each E–T distance the current was recorded and the difference in the current between the bulk media (sensor located > 1 mm away from the tissue) and that at various E–T distances were measured (Fig. 2B). The current was then plotted against each E–T distance (Fig. 2C). This resultant plot an exponential decay. The natural log of the current was plotted against the E–T distance and a linear regression line was fitted to the data enabling the slope and intercept to be derived (see Fig. 2D).

**Figure 3:**
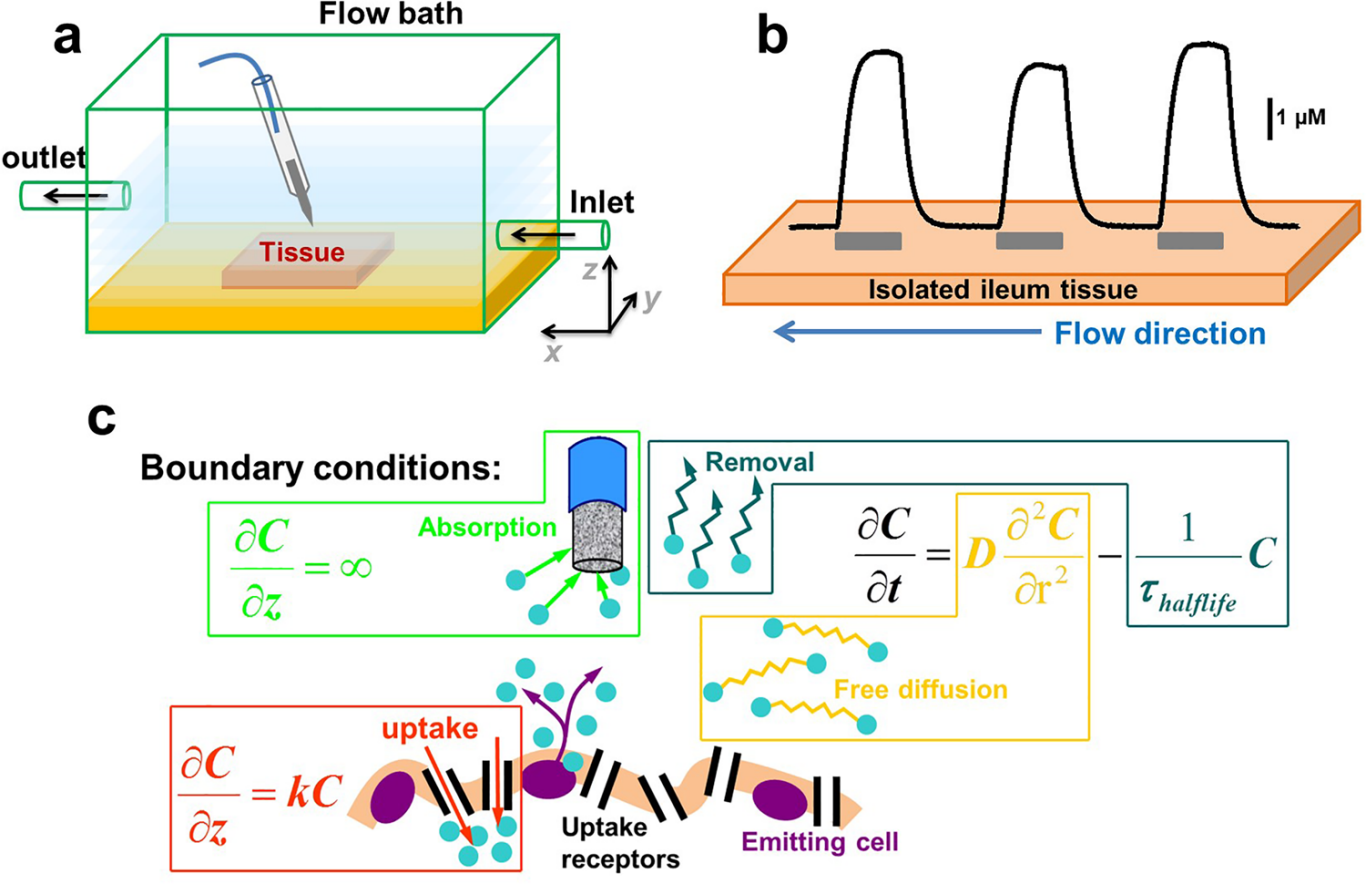
Experimental set and mathematical model utilised to define the parameters that could be monitored from amperometric approach curve profiling experiments. (**a**) Experimental conditions for isolated tissue measurements, where measurements were conducted in a rectangular flow bath with inflow and out in opposing directions. (**b**) Experimental responses obtained over varying regions of the tissue along the x plane. The grey boxes indicate when the electrode was placed at a E–T distance of 400 µm on isolated ileum tissue for a duration of 40 s. (**c**) The boundary conditions of the model show that there are four variables that are responsible for removal of extracellular 5-HT. These include oxidation at the electrode, removal due to convection and diffusion and finally removal by SERT following reuptake.

For interpretation of the results, it is useful to understand the experimental setup. Figure 3a shows a schematic diagram of the flow bath, where the inlet–outlet direction is marked as *x* direction, while the E–T direction is marked as *z* direction. If the buffer perfusion produced a laminar flow in the bath, the flow would generate a concentration gradient of 5-HT molecules in the *x* direction (lower concentration at the inlet, higher concentration in the outlet). Following our observations, the buffer perfusion produced a turbulent flow, instead; therefore no significant concentration gradient in the *x* direction was detected by measuring the concentration of 5-HT overflow at E–T distances of 400 µm at varying positions along the *x* direction (Fig. 3b). However, the turbulent flow can contribute to establishing a gradient in the *z* direction. In fact, such a flow could drive 5-HT molecules from the bulk to the tissue, exposing the molecules to the reuptake mechanisms, and acting effectively as a half-life/removal mechanism (Fig. 3c). It is known that a half-life/removal mechanism establishes a concentration gradient with an exponential profile. This ties in with our previous work^39^ in which we conducted Brownian dynamics simulations based on reaction–diffusion model^40^. The model shown in Fig. 3c considers the following boundary conditions: (1) all 5-HT molecules are released into the bathing solution from point sources representing the EC cells and from there they diffuse way, (2) all parts of the tissue other than the EC cell are responsible for reuptake of 5-HT (as the EC cell plays a minor role within reuptake relative to adjacent enterocyte cells), (3) all 5-HT molecules reaching the electrode are removed and counted. Under these conditions, our simulations show that the intercept of the exponential gradient provides a good marker of 5-HT release from EC cells and the slope correlates with 5-HT reuptake.

### Investigating primary and secondary regulatory processes from intestinal tissue

**Figure 4:**
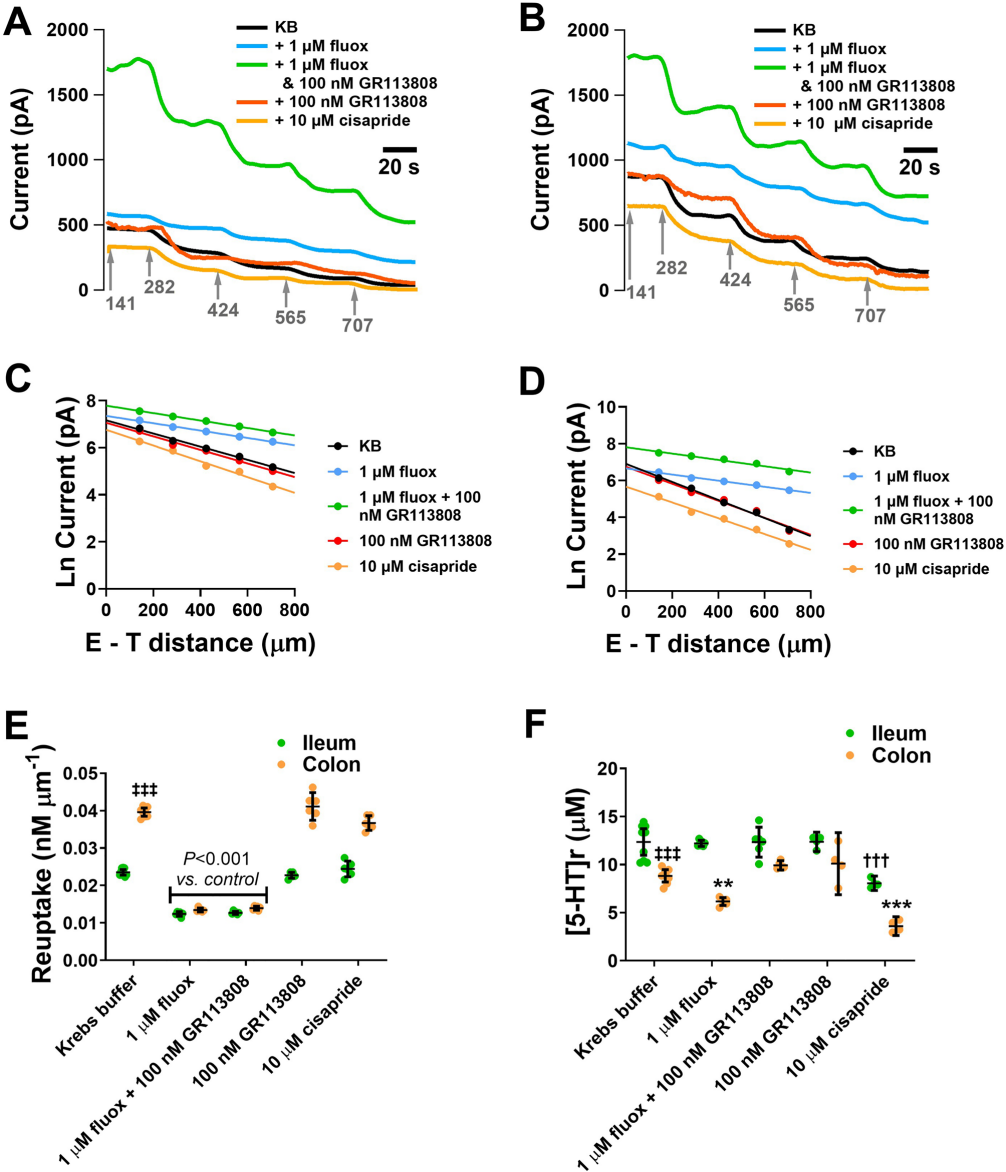
Amperometry approach curve profiling for studying the extracellular regulation of 5-HT release (**A**) Electrochemical responses from ileal mucosal tissue taken at varying E–T distances (grey numbers). Arrows indicate when the electrode is positioned at a new E–T distance. Distances are shown are in µm. All experiments are carried out at + 650 mV vs Ag|AgCl using a 76 µm BDD microelectrode. Experiments were performed in the presence of 1 µM Fluoxetine (Fluox), 1 µM Fluox and 100 nM GR113808, 100 nM GR113808 and 10 µM cisapride. (**B**) Electrochemical current responses from colonic mucosal tissue. (**C**) Plot showing natural log of current versus the E–T distance from ileum tissue. (**D**) Plots showing the natural log of current versus the E–T distance from colon tissue. (**E**) Changes in 5-HT reuptake. (**F**) Changes observed in 5-HT release ([5-HT]r) following a range of pharmacological interventions. Data shown as mean ± st.dev. (n = 4–9) ^‡‡‡^*p* < 0.001 ileum vs. colon; ^†††^*p* < 0.001 compared to Krebs buffer response in ileum and *p < 0.05, **p < 0.001 and ***p < 0.001 compared to Krebs buffer response in colon.

Experimental responses are shown in Fig. 4A and B. As the E–T distance increases, the current decreases in both ileum and colon tissue. When the natural log of the current is plotted against the E–T distance (Fig. 4C and D), a clear difference in the response between the ileum and colon can be observed. The slope under control conditions is far steeper in the colon (Fig. 3D) than the ileum (Fig. 4C) indicating that re-uptake is greater in the colon (*p* < 0.001, n = 6–9, Fig. 4E). There was also a significantly lower amount of 5-HT released from the colon compared to the ileum (*p* < 0.001, n = 8–9, Fig. 4F).

In the presence of 1 µM fluoxetine (SERT inhibitor) there was a significant decrease in the reuptake of both the ileum and colon tissue samples (*p* < 0.001, n = 6–8, Fig. 4E). These findings highlight our approach provides clear insight into 5-HT reuptake. However, there was no significant decrease in 5-HT release, in the presence of 1 µM fluoxetine in the ileum, but a significant difference was observed in the colon (*p* < 0.01, n = 6–8, Fig. 4F). Importantly, this significant decrease in release in the colon was reversed in the presence of 100 nM GR113808 (5-HT_4_ antagonist) and 1 µM fluoxetine (n = 5–8, Fig. 4F), suggestive that the fluoxetine-evoked decrease in 5-HT release was initiated by activation of an inhibitory 5-HT_4_ autoreceptor. Further confirmation of the presence of an inhibitory 5-HT_4_ autoreceptor was obtained by observing a decrease in the release of 5-HT in the presence of 10 µM cisapride (5-HT_4_ agonist) in the colon (*p* < 0.001, n = 4–8, Fig. 4F). Application of the 5-HT_4_ antagonist and agonist had no significant effect on re-uptake in either the ileum or the colon (Fig. 4E).

In the ileum the addition of 100 nM GR113808 had no effect on release when compared to control. However, application of the 5-HT_4_ agonist, cisapride was capable of significantly reducing release in the ileum (*p* < 0.001, n = 4–9, Fig. 4F).

### Effect of 5-HT selective reuptake inhibitors (SSRIs)

**Figure 5:**
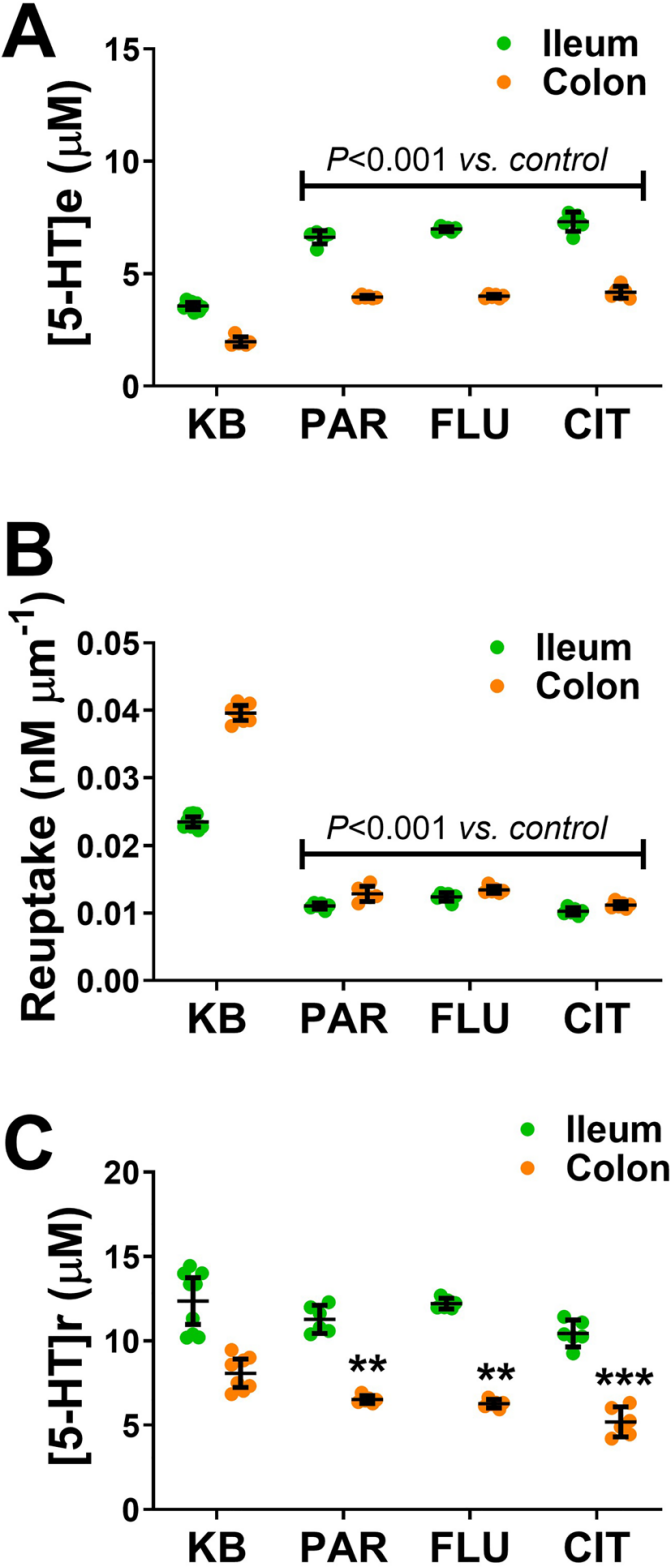
Influence of SSRIs. (**A**) Concentration of extracellular 5-HT ([5-HT]e) monitored at an E–T distance of 400 µm from both ileum and colon tissue. Measurements carried out in 1 µM fluoxetine (FLU), 1 µM paroxetine (PAR) and 1 µM citalopram (CIT). (**B**) Changes in 5-HT reuptake in the presence of the different SSRIs. (**C**) Changes in 5-HT release ([5-HT]r) in the presence of various SSRIs. Where **p < 0.01 and ***p < 0.001 vs. control (n = 6–9).

As shown in Fig. 4F the SERT inhibitor fluoxetine could decrease the release of 5-HT in the colon. Further studies were carried out to observe if this effect was observed with a range of different SERT inhibitors or was just secondary effect of fluoxetine. 5-HT extracellular concentration was elevated in the presence of the various SSRIs in both the ileum and colon (n = 6–9, *p* < 0.001, Fig. 5A). SSRIs are known to act by reducing extracellular 5-HT clearance, and when the slope is measured there was a significant decrease in the rate of re-uptake in both the ileum and colon in the presence of all three SSRIs (n = 6–9, *p* < 0.001, Fig. 5B). There was also a significant decrease in 5-HT release from the colon in the presence of the three SSRIs (n = 6–9, *p* < 0.01 for paroxetine and fluoxetine and *p* < 0.001 for citalopram, Fig. 5) There was no difference in the release of 5-HT from the ileum in the presence of the various SSRIs.

**Figure 6:**
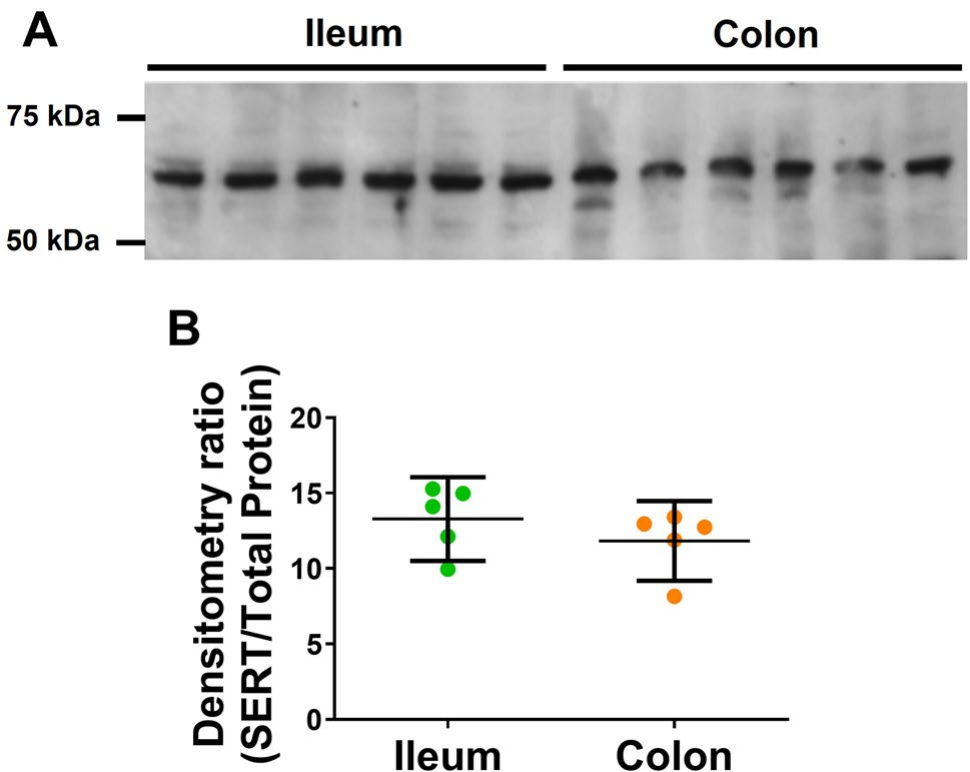
Western blot of SERT. (**A**) The anti-SERT antibody detected a major band at approximately 67 kDa. (**B**) Normalisation of the bands to the total protein load of the Coomassie-labelled membrane showed that there was no significant difference in the SERT content between the ileum and colon tissue (n = 5).

The rate of reuptake is greater in the colon compared to the ileum (Fig. 4E, *p* < 0.001), suggesting that there is either more SERT expression or greater activity of SERT in the colon. Figure 6 shows that there was no differences in SERT expression in the ileum and colon (n = 6–9, full length blot in Figure S1 ), which is suggestive that the greater rate of reuptake within the colon is most likely due to increased activity of SERT.

### Dose-response relationships for the regulatory mechanisms

Our data suggest that the presence of an inhibitory 5-HT_4_ autoreceptor can regulate 5-HT release in the colon. To understand the sensitivity of the 5-HT_4_ inhibitory autoreceptor, and SERT transporters to extracellular levels of 5-HT, amperometry approach curve profiling was carried out in the presence of varying concentrations of fluoxetine as an indirect means of elevating extracellular 5-HT levels. Clear changes in the slope and intercept were observed from colon tissue perfused with fluoxetine concentrations ranging from 200 nM to 2 µM (Figure S2).

**Figure 7:**
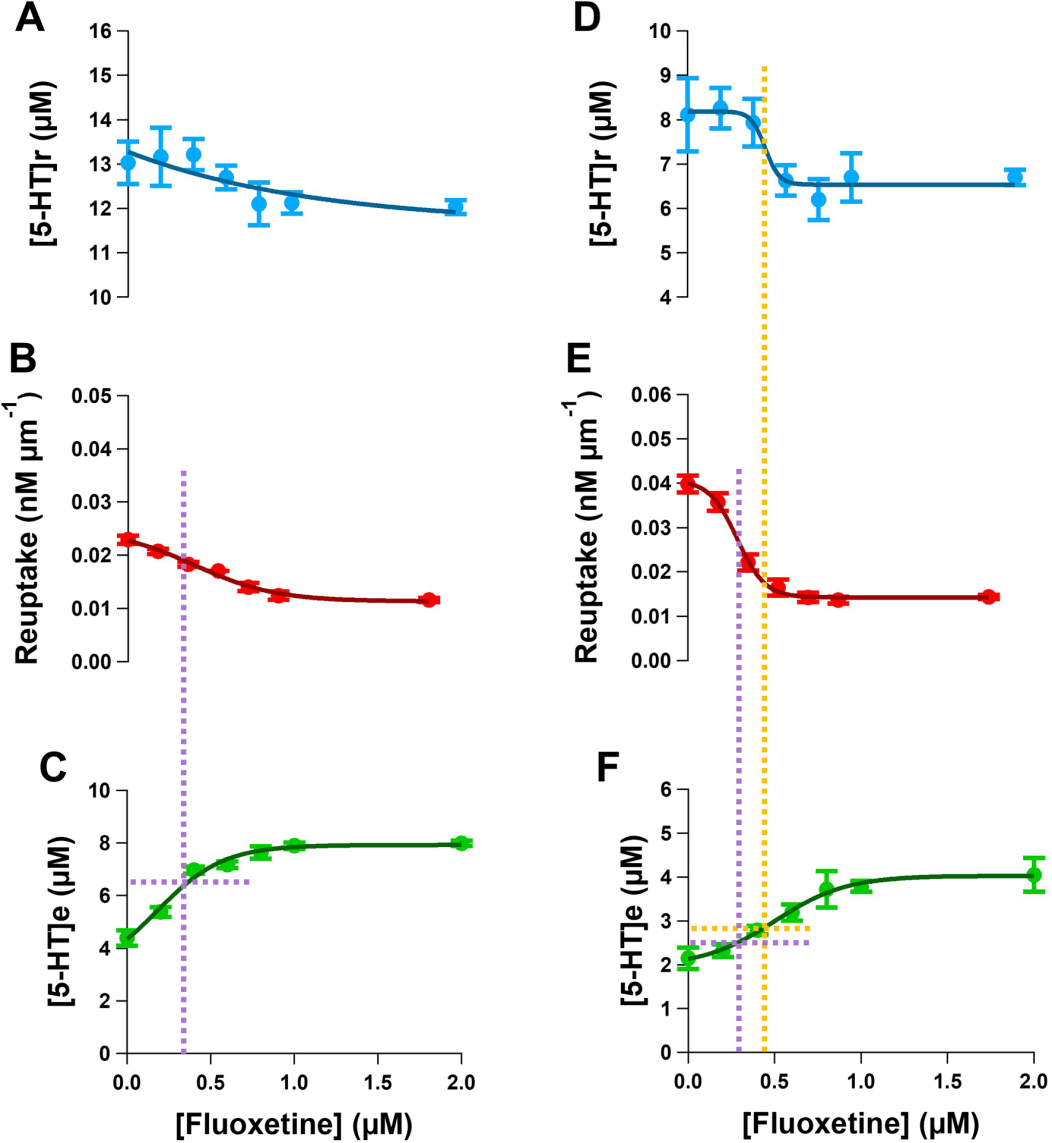
Amperometry approach curve profiling to unravel the properties of the SERT and autoreceptors. Current approach curve profiles were obtained at various concentrations of fluoxetine. (**A**) Release of 5-HT ([5-HT]r), (**B**) reuptake of 5-HT and (**C**) extracellular concentration of 5-HT ([5-HT]e) in the ileum. (**D**) Release of 5-HT ([5-HT]r), (**E**) reuptake of 5-HT and (**F**) extracellular concentration of 5-HT ([5-HT]e) in the colon. (n = 6) The dotted orange line indicates the concentration of fluoxetine that causes a 50% reduction in 5-HT release. The dotted purple line indicated the IC_50_ for fluoxetine at the SERT.

Release, re-uptake and the extracellular 5-HT concentration were plotted against increasing concentrations of fluoxetine for both the ileum and colon tissue (Fig. 7). Amperometry approach curve profiling allows a comparison across the three variables simultaneously allowing for an analysis to be made about their contribution to extracellular 5-HT concentration. 5-HT release was not altered significantly in the ileum with increasing concentrations of fluoxetine. However, 5-HT reuptake was reduced with a consequential increase in the extracellular 5-HT concentration (n = 6, Fig. 7aii and 7aiii). Together these data suggest that inhibitory 5-HT autoreceptors are not likely to be regulating 5-HT release in the ileum mucosa under our experimental conditions. From the resultant graphs the half maximal inhibitory concentration (IC_50_) of the SERT transporter was achieved using 0.43 ± 0.08 µM fluoxetine.

For the colon, 5-HT release decreased with increasing concentrations of fluoxetine (Fig. 7bi), which indicates the activation of inhibitory 5-HT_4_ autoreceptors, as this response can be reversed with the addition of 100 nM GR113808, a selective 5-HT_4_ receptor antagonist (Fig. 4E). The IC_50_ for inhibiting 5-HT release, presumable through the activation of the 5-HT_4_ autoreceptors was observed in the presence of 0.46 ± 0.05 µM fluoxetine which equates to 2.9 ± 0.12 µM of 5-HT. Activation of 50% of the colonic inhibitory 5-HT_4_ autoreceptors requires a 40% increase in extracellular 5-HT concentration (n = 6, Fig. 7b). The IC_50_ for the SERT transporter can also be gained from the same experiment. Our study indicates the IC_50_ for the SERT transporter is 0.32 ± 0.04 µM fluoxetine (n = 6, Fig. 7bii). There was no significant difference in the IC_50_ for the SERT transporter between ileum and colon tissues. Within our studies the IC_50_ obtained for SERT transporter from the ileum and colon were within a range published in other studies^41–43^. This also shows and validates that are technique is suitable for generating dose response curves for monitoring pharmacology parameters.

## Discussion

This study has shown that the technique of amperometry approach curve profiling can provide the means to understand more about the regulatory processes that govern the extracellular concentration of 5-HT in the ileum and colon. Specifically, this novel approach provides: (i) an ability to understand the contribution of release and reuptake to extracellular transmitter concentrations using a single series of current measurements at various E–T distances, (ii) provides an ability to investigate the dose–response relationships of the transporter and autoreceptor and (iii) an ability to determine the sensitivity of the autoreceptors to and transporter to endogenous 5-HT. This technique therefore provides a way of profiling the mechanisms that regulate extracellular 5-HT concentrations in the GI tract and could potentially be used to determine which processes are altered during a range of disease states.

### Suitability of amperometry approach curve profiling for monitoring regulatory mechanisms that govern the extracellular concentration of 5-HT

Our amperometry approach curve profiling provides the data required to understand the contribution that release, reuptake and autoreceptors play in regulating the transmission process. Within our approach we have used the intercept as a measure of the amount of 5-HT being released from the EC cells and the slope as a measure of the reuptake of 5-HT by SERT. These two parameters provide a suitable estimate of these biological processes as this was based on the results of a Brownian dynamics model^40,44^. However, such suitability can also be validated through bath perfused pharmacological investigations. In Fig. 4 we have shown that in the presence of the SSRI fluoxetine, the slope, a marker of reuptake is mainly influenced, with no effect on the intercept, a marker of 5-HT release. This is especially true in the case of the ileum where autoreceptors are either not activated or present. This result was shown for various SSRIs in Fig. 5. Support for the intercept being a marker of 5-HT release comes through the response of the tissue to the application of the 5-HT_4_ receptor agonist (10 µM cisapride) and antagonist (100 nM GR113808). Both these pharmacological agents will affect the function of autoreceptors present on the EC cell and therefore will only influence the release of 5-HT from EC cells without affecting re-uptake. These ideas are supported by the data presented in Fig. 4E and F where the agonist and antagonist for the 5-HT_4_ autoreceptor only influenced the release component. Overall, our study indicated that the slope and intercept make good predictors of transmitter reuptake and release respectively.

Although this approach offers good insight into the mechanisms that govern extracellular concentration of the transmitter, this approach is only suitable in tissues where steady state conditions are observed as opposed to dynamic signalling changes observed in the nervous system. This is the case in the ex vivo ileum or colon due to the constant mechanosensory stimulation of the EC cells evoked by the perfusion of the Krebs buffer. This approach also provides good insight into the presence of inhibitory autoreceptors but is limited in understanding the activity of excitatory autoreceptors due to a reduced ability to reduce 5-HT release in real time. This methodology is also only suitable in gaining information on a segment of tissue, rather than providing spatial knowledge of signalling.

Although the experimental data supports the model for both release (intercept) and reuptake (slope), the approach all must be used with some caution as the experimental conditions are far more complex that than of our mathematical model. One of the major limitations is the accuracy of the data generated when the geometry of the measurement environment is so varied between the ileum and colon. Our model is best suited to the colon, which is formed of relatively flat columnar epithelium held together by tight junctions, when compared to the large individual villi of the ileum ≈ 300 µm length). It is highly likely that the release is underestimated in the ileum when compared to the colon, as release of 5-HT can occur from EC cells located in the crypts of the villi and thus only a degree of this release may be observed extracellularly.

Overall, this model shows the ability to monitor release and reuptake from the ileum and colon and has the potential to provide insight into signalling mechanisms during a range of pathophysiological conditions.

### Amperometry approach curve profiling versus other approaches for monitoring regulatory mechanisms

In areas where steady state basal level of signalling molecules is observed, there are no studies that unravel reuptake and release from a single electrochemical measurement without the need of pharmacological agents. The vast number of amperometric or FSCV studies conducted to-date from single cells to in vivo in various brain regions monitor either spontaneous or stimulate signals, which have dynamic features and thus enable the ability to gain insight into release and reuptake process^45,46^. In this aspect amperometry approach curve profiling offers a major advantage as it provides a methodology which can decouple release and reuptake from basal changes in the current. The approach is simple and are therefore useful to non-specialists in the field.

Additionally, our technique provides the ability to understand the contribution of autoregulation to the extracellular transmitter concentration. Current techniques for measuring the contribution that autoreceptors make to the transmission process require using agonists and antagonists of the autoreceptor during various analytical measurements^16,47^. The major limitation of this approach is that the activity of the natural ligand, in our case 5-HT, on the autoreceptor cannot be fully understood. This is not a problem with amperometry approach curve profiling as we are able to both record the activation of the autoreceptor and the extracellular 5-HT concentration from a single measurement, enabling the results to be directly compared. The use of fluoxetine as an indirect means of elevating 5-HT levels provides a novel approach to understand how changes in extracellular 5-HT levels affects inhibitory autoreceptors and the precise concentrations of 5-HT that regulate these receptors.

### Differences in the regulatory mechanisms between ileum and colon

Our study clearly demonstrates significant differences in the transmitter regulatory processes used to regulate the extracellular 5-HT concentration in the ileum and colon. Both SERT and 5-HT_4_ inhibitory autoreceptors are active in colonic tissue whilst only SERT serves to regulate increases in extracellular concentrations of 5-HT in the ileum. This strongly suggests that the extracellular 5-HT concentrations in the colon are more tightly regulated than that within the ileum. The IC_50_ of SERT to fluoxetine and the Western Blot expression of SERT protein were the same in both the ileum and colon tissues. As the rate of reuptake was greater in the colon than the ileum, these findings suggestive that the activity of SERT in the colon is greater than that in the ileum. Because of all these factors, the extracellular concentration of 5-HT in the colon is significantly lower than that in the ileum. This observation is similar to observations in previous studies, where the concentration of luminal 5-HT has been shown to decrease as you go down the intestinal tract^17,39^.

Our findings identify the activity of an inhibitory 5-HT_4_ autoreceptor on murine colonic EC cells. Other studies have shown the presence of 5-HT_4_ autoreceptors on the EC in various animal species^1,14,16,48^. Therefore, there is no debate on the presence of 5-HT autoreceptors on the EC cell, but the type of autoreceptor and its effect on transmitter release varies depending on intestinal location and species. In this study the role of the 5-HT_4_ autoreceptors has been shown to be inhibitory within the colon.

The various regulatory processes (release, reuptake and autoregulation) in the colon can rapidly activated to regulate extracellular 5-HT to allowing the colon to control motility to maximise the amount of water is absorbed from the faecal matter and optimise waste removal. This tight regulation of extracellular 5-HT is less likely in the ileum indicative that motility patterns are less variable in this region of the bowel.

## Conclusion

Amperometry approach curve profiling provides a simple and rapid approach for understanding the concentration profile of oxidisable substances in the vicinity of the tissue, which in turn can provide information on the release and reuptake of transmitters. Combining the method with the use of pharmacological agents provides a novel way of understanding the dose–response relationships of autoreceptor and transporters. This technique therefore provides a more in-depth examination of the transmission process using a simple series of measurements that limits the use of tissue and helps to unravel how mechanisms that regulate extracellular transmitter concentrations may alter in various pathophysiology conditions.

## Methods

### Ethical approval

All procedures were carried out according to U.K. Home Office regulations and were approved by the University of Brighton Ethics Committee. All experimental protocols conducted within the study was performance in accordance with ARRIVE guidelines. Male C57BL/6 J mice were obtained from Harlan UK at 8 weeks of age and kept under a conventional housing system. Animals were maintained at 20.0 ± 2 °C, 50 ± 5% humidity and fed on a maintenance diet (R&M diet no1 SDS expanded diet) until required. Animals aged 3 months were euthanized using carbon dioxide and exsanguinated following cervical decapitation. For the distal ileum segments, 2 cm sections of tissue were taken 2 cm proximal from the caecum and the distal part of the colon 2 cm proximal to the anus and both were placed in ice cold oxygenated (95% O_2_ and 5% CO_2_) Krebs’ buffer solution, pH 7.4 (117 mM NaCl, 4.7 mM KCl, 2.5 mM CaCl_2_, 1.2 mM MgCl_2_, 1.2 mM NaH_2_PO_4_, 25 mM NaHCO_3_, and 11 mM glucose).

### Amperometry approach curve profiling recordings

For approach curve profiling measurements, constant potential amperometry was utilised. A platinum wire was used as the auxiliary electrode and a “no leak” Ag|AgCl electrode (3 M KCl, Cypress Systems Inc., USA) served as the reference electrode in a three-system configuration. The working electrode consisted of a 76 µm boron-doped diamond (BDD) microelectrode. Details on the construction and performance characteristics of the BDD microelectrodes have been previously published^19^. All the electrodes were placed in a flow bath. The flow bath was mounted on the stage of an inverted microscope (Accu-Scope, USA) and continuously perfused with warm (37 °C) oxygenated Krebs’ buffer solution at a flow rate of 4 ml min^−1^. Ileum or colon segments were placed into the Krebs’ buffer solution prior to measurements. The segments were bisected along the mesenteric border to open the preparations and the tissue was pinned down with the mucosal layer upmost. Tissues were left for 30 min prior to commencing recordings.

For measurements, the BDD microelectrode was affixed to a micromanipulator (Fine Scientific Tools, USA) and placed > 5 mm over the centre of a tissue piece in the bulk of the media. The BDD electrode was held at a potential of + 650 mV *vs.* Ag|AgCl. During measurements, the electrode was carefully positioned over the tissue for a duration of 40 s for each of the following E–T distances: 1000, 800, 600, 400 and 200 µm (see Fig. 1). These distances were chosen based on a previous study that investigated the error associated with monitoring at various distances^39^. This multi-step approach curve profile was repeated over two or three regions of the same tissue section. Following control measurements in each tissue, measurements were carried out in the same tissue sample to assess the influence of various pharmacological agents. These included the 5-HT selective reuptake inhibitors: fluoxetine (concentrations ranging 200 nM to 2 µM), citalopram (1 µM) and paroxetine (1 µM); 5-HT_4_ receptor antagonist GR113808 (100 nM) and 5-HT_4_ receptor agonist cisapride (1 µM). All drugs were obtained from R&D Systems, Abingdon, UK.

### Western Blot analysis for determination of SERT content

Colon and ileum segments from 3-month-old mice were placed in chilled Krebs’ solution, bisected along the mesenteric border to expose the mucosal tissue, which was then scraped away and snap frozen in liquid N_2_ for storage. Tissue was placed on ice in lysis buffer (10 mM HEPES, 150 mM NaCl, 1 mM EDTA, 0.2% Nonidet P40, protease inhibitor cocktail P8340, Sigma-Aldrich Inc.) and manually lysed. Lysates were centrifuged for 10 min, at 4 °C at 700 × *g* to pellet debris and nuclear components. Supernatants were removed and their protein content assessed using Quick Start Bradford Dye reagent (Bio-Rad). 15 µg of protein from each sample was combined with an equal volume of 2 × Laemmli loading buffer (S3401, Sigma Aldrich), separated on a 10% SDS-PAGE gel using the Mini-protean II electrophoresis cell (Bio-Rad) and transferred to Immobilon polyvinylidene fluoride (PVDF) membrane using the Trans-Blot® wet transfer (Bio-Rad). Membranes were blocked with 5% milk in PBS-Tween 20 (0.2%) for 3 h and then incubated overnight at 4 °C with a rabbit anti-SERT antibody (#24330, Immunostar Inc.) diluted to 1:1500 in milk-PBS-Tween 20. Membranes were washed three times in PBS-Tween 20 and then incubated with goat anti-rabbit HRP-conjugated secondary antibody at 1:2000 (SC-2005, Santa Cruz Biotechnology) for 1 h at room temperature. After five further washes with PBS-Tween 20, membranes were treated with Amersham™ ECL plus western blotting detection system (GE Healthcare) and exposed to Amersham™ ECL plus film. The relative intensity of each SERT band was measured by densitometry using the Fluorochem™ imager (Alpha Innotech) and the signal was normalised to the total protein in that lane, determined by staining the PVDF membrane with Coommassie Brilliant Blue (Bio-Rad).

### Data analysis and interpretation

For amperometric approach curve profiling measurements the differences in the current between the bulk media (sensor located > 1 mm away from the tissue) and that at various distances of interest were obtained for each E–T distance. The current was then plotted against each E–T distance. The natural log of the current in whole numbers (pA scale) was calculated and plotted against the E–T distance and a linear regression line was fitted to the data enabling the slope and intercept to be derived (see Fig. 1). Based on the mathematical modelling (Fig. 2), the slope (measure of 5-HT reuptake) and intercept (measure of 5-HT release) were obtained. Finally, the current responses were converted to concentrations of 5-HT using calibration plots. The results for ileum and colon tissue and the differences between control measurements and those in the presence of various pharmacological agents were compared using either a 2-way or 1-way ANOVA test with a Bonferroni post-test.

### Supplementary Information

Supplementary Information.

## Data Availability

The datasets used and analysed during the current study available from the corresponding author on reasonable request.
