# Supplementary material for: Amperometry approach curve profiling to understand the regulatory mechanisms governing the concentration of intestinal extracellular serotonin
Source: Sci Rep. 2024 May 7;14:10479. doi: 10.1038/s41598-024-61296-9 (PMC11076564; doi:10.1038/s41598-024-61296-9)
Supplement: Supplementary file 1 — Supplementary Information. [file 41598_2024_61296_MOESM1_ESM.docx]

**Supporting information**

**Amperometry approach curve profiling to understand the regulatory mechanisms governing the concentration of intestinal extracellular serotonin**

Mark S. Yeoman^1,2^ (ORCID ID: 0000-0003-2113-8650), Sara Fidalgo^1,2^, Gianluca Marcelli^3^ (ORCID ID: 0000-0002-7475-7327) and Bhavik Anil Patel^1,2^* (ORCID ID: 0000-0002-8773-3850)

^1^School of Applied Sciences and ^2^Centre for Lifelong Health, University of Brighton, Huxley Building, Brighton, BN2 4GJ

^3^School of Engineering, University of Kent, Jennison Building, Canterbury, CT2 7NZ


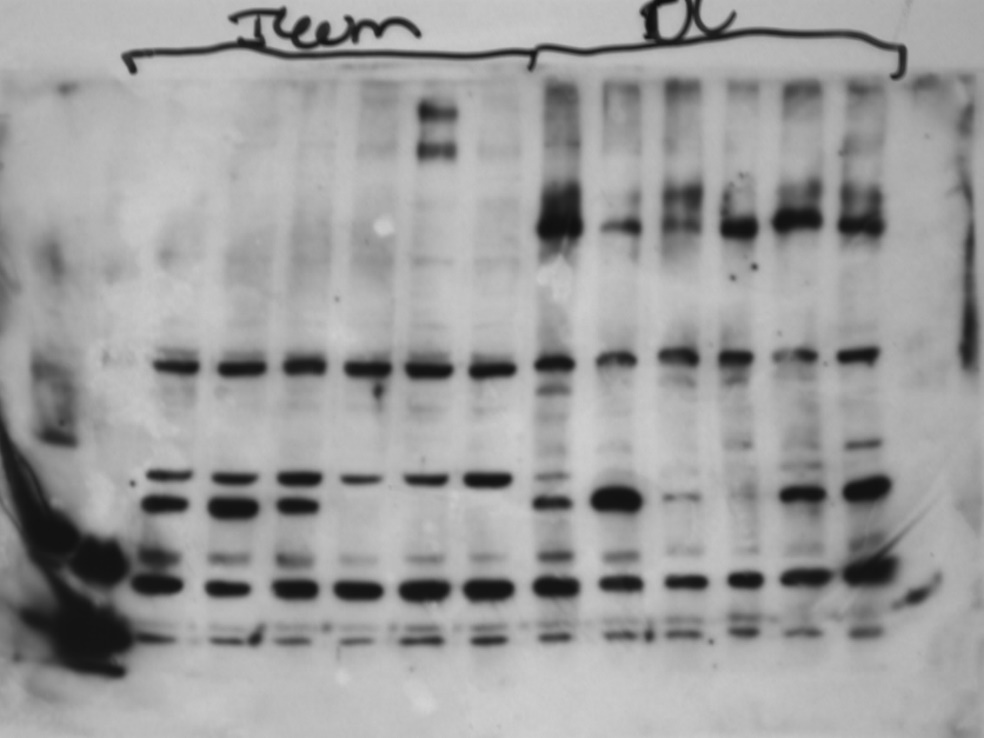


**Figure S1.** Full length Western blot to investigate the expression of SERT in ileum and colon tissue

**Figure S2.** Plot showing natural log of current versus the E-T distance from colon tissue in the presence of varying concentrations of fluoxetine (ranging from 200 nM to 2 µM).
